# Exome and Tissue-Associated Microbiota as Predictive Markers of Response to Neoadjuvant Treatment in Locally Advanced Rectal Cancer

**DOI:** 10.3389/fonc.2022.809441

**Published:** 2022-03-22

**Authors:** Isabella Kuniko T. M. Takenaka, Thais F. Bartelli, Alexandre Defelicibus, Juan M. Sendoya, Mariano Golubicki, Juan Robbio, Marianna S. Serpa, Gabriela P. Branco, Luana B. C. Santos, Laura C. L. Claro, Gabriel Oliveira dos Santos, Bruna E. C. Kupper, Israel T. da Silva, Andrea S. Llera, Celso A. L. de Mello, Rachel P. Riechelmann, Emmanuel Dias-Neto, Soledad Iseas, Samuel Aguiar, Diana Noronha Nunes

**Affiliations:** ^1^ Medical Genomics Laboratory, International Center for Research, A.C.Camargo Cancer Center, São Paulo, Brazil; ^2^ Laboratory of Bioinformatics and Computational Biology, International Center for Research, A.C. Camargo Cancer Center, São Paulo, Brazil; ^3^ Laboratorio de Terapia Molecular y Celular – Genomics Unit, Fundación Instituto Leloir, Buenos Aires, Argentina; ^4^ Instituto de Investigaciones Bioquímicas de Buenos Aires (IIBBA), Consejo Nacional de Investigaciones Científicas y Técnicas (CONICET), Buenos Aires, Argentina; ^5^ Oncology Unit, Hospital de Gastroenterología Carlos Bonorino Udaondo, Buenos Aires, Argentina; ^6^ Clinical Oncology, Intergrupo Argentino para el Tratamiento de los Tumores Gastrointestinales (IATTGI), Buenos Aires, Argentina; ^7^ Department of Pathology, A.C.Camargo Cancer Center, São Paulo, Brazil; ^8^ Colorectal Cancer Department, A.C.Camargo Cancer Center, São Paulo, Brazil; ^9^ Department of Clinical Oncology, A.C.Camargo Cancer Center, São Paulo, Brazil; ^10^ Laboratory of Neurosciences (LIM-27) Alzira Denise Hertzog Silva, Institute of Psychiatry, Faculdade de Medicina, Universidade de São Paulo, São Paulo, Brazil; ^11^ National Institute of Science and Technology in Oncogenomics and Therapeutic Innovation (INCITO), São Paulo, Brazil

**Keywords:** locally advanced rectal cancer, neoadjuvant chemoradiotherapy, whole exome sequencing, microbiota, biomarkers of treatment response, mutational signatures

## Abstract

The clinical and pathological responses to multimodal neoadjuvant therapy in locally advanced rectal cancers (LARCs) remain unpredictable, and robust biomarkers are still lacking. Recent studies have shown that tumors present somatic molecular alterations related to better treatment response, and it is also clear that tumor-associated bacteria are modulators of chemotherapy and immunotherapy efficacy, therefore having implications for long-term survivorship and a good potential as the biomarkers of outcome. Here, we performed whole exome sequencing and 16S ribosomal RNA (rRNA) amplicon sequencing from 44 pre-treatment LARC biopsies from Argentinian and Brazilian patients, treated with neoadjuvant chemoradiotherapy or total neoadjuvant treatment, searching for predictive biomarkers of response (responders, *n* = 17; non-responders, *n* = 27). In general, the somatic landscape of LARC was not capable to predict a response; however, a significant enrichment in mutational signature SBS5 was observed in non-responders (*p* = 0.0021), as well as the co-occurrence of *APC* and *FAT4* mutations (*p* < 0.05). Microbiota studies revealed a similar alpha and beta diversity of bacteria between response groups. Yet, the linear discriminant analysis (LDA) of effect size indicated an enrichment of *Hungatella, Flavonifractor*, and *Methanosphaera* (LDA score ≥3) in the pre-treatment biopsies of responders, while non-responders had a higher abundance of *Enhydrobacter, Paraprevotella* (LDA score ≥3) and *Finegoldia* (LDA score ≥4). Altogether, the evaluation of these biomarkers in pre-treatment biopsies could eventually predict a neoadjuvant treatment response, while in post-treatment samples, it could help in guiding non-operative treatment strategies.

## 1 Introduction

Locally advanced rectal cancers (LARCs) constitute one-third of all colorectal tumors and present a well-established treatment, comprising two standardized protocols. One strategy is the intravenous or oral administration of 5-fluorouracil (5-FU)-based neoadjuvant chemoradiotherapy (nCRT), followed by surgery, while the other is the total neoadjuvant treatment (TNT), which delivers both fluorouracil- and oxaliplatin-based chemotherapy and chemoradiotherapy prior to surgery. Despite the recent advances in the management of LARC, the responses to multimodal neoadjuvant therapy (chemoradiation) vary widely among patients. Pathological complete response (pCR) is defined as the absence of viable tumor cells in the surgical resection specimen and occurs in approximately 10%–30% of patients treated with nCRT, reaching between 17.2% and 38.5% in LARC-patients treated with TNT (1–5). Whereas previous studies have shown pCR to be an important prognostic factor for overall survival (OS) (6, 7) non-responder (NR) patients, instead of having their tumors surgically removed right after diagnosis, are otherwise exposed to the toxic effects of a non-effective chemoradiation (6, 8). Therefore, the identification of predictive biomarkers of complete response before treatment could be very beneficial for the management of LARC patients.

Several studies have evaluated the importance of clinical and pathological markers potentially associated with nCRT response. For instance, the pathological grade, tumor size, clinical stage determined by imaging techniques, pre-treatment levels of the carcinoembryonic antigen (CEA), nCRT and surgery intervals, and tumor budding, among others, may impact the nCRT response (1, 9–11). More recently, molecular approaches such as the identification of gene mutations, gene expression profiles (12), genomic instability (13), and DNA methylation (14) have been evaluated in pCR prediction and some frequently mutated genes were identified (15). However, some findings are still controversial and depend on validation in larger independent cohorts with a systematic and standardized pCR evaluation. Therefore, no biomarkers are currently used in the clinical setting (16–19). Overall, despite numerous efforts, the predictive markers for pCR in locally advanced rectal cancer with sufficient sensitivity and specificity are still lacking.

In the last few years, some groups have suggested that not only the tissue-associated microbiota composition is significantly different between rectal cancer and non-cancer samples (20) but also that tumor-associated bacteria are directly related to the efficacy of chemotherapy and immunotherapy in melanoma, lung, and pancreatic cancers (21–23). Moreover, Riquelme et al. (24) reported that the microbiota of pancreatic tumors influences long-term survival in patients with resected pancreatic ductal adenocarcinoma (PDAC), although the predictive role of microbiota in response to cancer-directed therapies remain undetermined. Furthermore, a recent study showed that bacteria are associated with seven distinct tumor types, where they commonly have intracellular locations in tumors and in some immune cells (25).

*Fusobacterium nucleatum* is a well-known gut bacterium extensively associated with pre-neoplastic lesions in colonic mucosae, colorectal tumors, and colorectal tumor recurrence (26–28). Although an increased abundance of *F. nucleatum* has been reported in rectal cancer patients with poor response to nCRT (26, 29), this species has not been confirmed as a universal marker of poor response. In this sense, due to the multifactorial nature of the neoplastic disease, it is likely that only a combination of different biomarkers will allow the development of sensitive and robust tests capable of identifying patients more likely to benefit from nCRT or TNT and achieve pCR.

In an attempt to contribute to the search for more robust biomarkers of treatment response in LARC, we present here a combined and prospective evaluation of tumor tissue-associated microbiota and whole exome sequencing (WES) from a cohort of 44 patients from Argentina and Brazil, diagnosed with LARC and treated with neoadjuvant therapy (chemoradiation plus/minus chemotherapy).

## 2 Material and Methods

### 2.1 Patients and Collection of Samples

Biopsies were collected prior to nCRT/TNT from patients that underwent colonoscopy examination for rectal cancer diagnosis between 2018 and 2020 at the A.C.Camargo Cancer Center (ACCCC), São Paulo, Brazil (*n* = 26) and Hospital de Gastroenterología Dr. Carlos Bonorino Udaondo, Buenos Aires, Argentina (*n* = 18). All Brazilian (BR) and Argentinian (AR) fresh-frozen tumor biopsy samples were stored at -80°C until further processing and slides from all samples were histologically examined to confirm the diagnosis of rectal cancer. LARC patients were prospectively recruited in this observational and multicentric study. Inclusion criteria were patients with: (i) histologically confirmed rectal adenocarcinoma and age >18 years old; (ii) candidates to initiate nCRT treatment with continuous infusion of 5-FU (fluorouracil) or oral capecitabine (825 mg/m^2^/twice a day), and radiotherapy (a total dose of 50.4 Gy in 28 fractions); (iii) or TNT treated patients (exclusively from Argentina) receiving induction treatment with three cycles of CAPOX (130 mg/m^2^ of oxaliplatin on day 1 and capecitabine 1,000 mg/m^2^/twice a day, for 14 days, every 3 weeks), followed by conventional nCRT. The classification of patients as responders (R) or NR was determined after the histopathological analysis of tissue samples collected during surgery. Our efforts were taken in order to match the patients from these groups according to gender, age, tumor location, and stage.

### 2.2 Response Evaluation

The assessment of treatment response was performed 8–12 weeks after completing radiotherapy by digital rectal exam (DRE), colonoscopy examination with biopsy collection, and imaging tests (thorax abdomen computed tomography [CT] and pelvic magnetic resonance imaging [MRI]), followed or not by surgery and adjuvant chemotherapy. From now on, nCRT and TNT will be referred to as nCRT throughout this manuscript. The responses to nCRT were defined according to the presence (pathological incomplete response, group NR, *n =* 27) or the absence (ypT0N0—responder patients, group R, *n =* 17) of reminiscent viable tumor cells in the surgical specimens. Patients classified as clinical complete responders by DRE, colonoscopy, CT, and MRI managed by a watch-and-wait protocol were assigned to the group R if there was a clinical and radiological/proctoscopy complete response. Patients were also classified according to the pathological tumor regression observed in the surgical specimen using the Protocol for the Examination of Specimens from Patients with Primary Carcinoma of the Colon and Rectum (v.4.0.1.0), as recommended by the College of American Pathologists (CAP) (30).

This study was approved by the ACCCC Review Board (2446/17), by the Udaondo Hospital Ethics Committee (HBU-ONCO-DEGENS) and the Instituto Leloir Institutional Review Board CBFIL (CBFIL#20, May/2015). All patients have voluntarily chosen to participate by signing an informed consent form prior to sample collection.

### 2.3 Whole Exome Sequencing and Analyses

WES was performed for R (*n* = 17) and NR patients (*n* = 27) after DNA extraction from tissue biopsies, collected at diagnosis (AllPrep DNA/RNA Mini Kit (Qiagen, Hilden, Germany). Tissues had an average 60% of tumor cells (all samples presented at least 30% of neoplastic cells detected by histological analysis). Two hundred nanograms of DNA were used for the construction of libraries (Agilent SureSelect Human All Exon v6 kit; Agilent Technologies, Santa Clara, CA, United States) and sequencing was performed on NextSeq 4000 (Illumina, USA) to generate paired end reads (2 × 100 bp), with at least 50× average vertical coverage (Macrogen, Seoul, South Korea). Sequencing reads were aligned to the GRCh38 human reference genome using the Burrows-Wheeler Alignment - Maximal Exact Match (BWA-MEM) (31), and all pre-processing steps were performed in accordance with the best practice guidelines of the Genome Analysis Toolkit (GATK4) (32). Duplicated reads were removed using Picard (v2.22.8; https://broadinstitute.github.io/picard/), base scores were recalibrated, and single-nucleotide variations (SNVs) and insertions/deletions were called with MuTect2 (v.4.1.7). In addition to the GATK4 hard filters, variants were filtered according to coverage, keeping only those confirmed by at least 5 altered reads in regions with >15× coverage, and with allele frequencies between 0.05 and 0.35 and frequencies ≤1% in non-cancer databases such as ExAC and gnomAD (https://gnomad.broadinstitute.org/), and 1000G databases (33).

In order to exclude potential germline variants, we excluded variants present in AbraOM [cohort SABE609, http://abraom.ib.usp.br/ (34)] and in our WES-panel of non-cancer BR subjects (*n =* 169) (data not published). Further analyses [tumor mutation burden (TMB), intratumoral heterogeneity (ITH), oncogenic pathways, and co-occurrent mutations] were performed using R packages maftools (v.2.8.05) (35) and ggplot2 (v.3.3.5) (36). Finally, mutational signatures were analyzed with signeR (37).

### 2.4 16S rRNA Gene Amplification, Sequencing and Bioinformatic Analyses

#### 2.4.1 DNA Extraction, PCR Amplification, and Sequencing

Fifty nanograms of genomic DNA from all fresh-frozen biopsies were used to generate amplicons to evaluate the microbiota (16S rRNA V3–V4 region). Amplicons were produced in 35 µl volume reactions containing 17.5 µl of KAPA2G Robust HotStart ReadyMix (KAPA Biosystems; Sigma-Aldrich, San Luis, MO, United States), template DNA and 5 µM of each oligonucleotide primer (Illumina sequencing adapters in bold): U341F (5’-CACTCTTTCCCTACACGACGCTCTTCCGATCT**CCTACGGGRSGCAGCAG**-3’), and 806R (5’-GTGACTGGAGTTCAGACGTGTGCTCTTCCGATCT**GGACTACHVGGGTWTCTAAT**-3’). The PCR amplification cycle consisted of an initial heating step of 95°C for 2 min, followed by 30 cycles of 95°C for 20 s and 54°C annealing for 15 s, and a final elongation step of 5 min at 72°C. Amplicons were purified with Ampure XP Beads (Beckman Coulter, Brea, CA, United States) and quantified by Qubit dsDNA High Sensitivity (Thermo Fisher Scientific, Waltham, MA, United States). A second PCR amplification was performed in triplicates to insert barcodes to the amplicons before sequencing, using 5 ng of template and a reaction mix with Taq Platinum (Invitrogen, Waltham, MA, United States). This PCR amplification step consisted of an initial heating step of 95°C for 5 min, followed by 10 cycles of 95°C for 45 s, 66°C for 30 s, 72°C for 45 s, and a final elongation step of 2 min at 72°C. Library triplicates were purified with Ampure XP Beads (Beckman Coulter, Brea, CA, United States) and pooled, followed by a quantification step by real-time PCR (KAPA Library Quantification Kit for Illumina Platforms—KAPA Biosystems, Sigma-Aldrich, San Luis, MO, United States). Sequencing was performed in the MiSeq platform (Illumina, United States) using MiSeq Reagent v2 (500-cycles) in paired-end mode.

#### 2.4.2 Microbiome Sequencing Analyses

As a preprocessing step, adapters and primers were trimmed using Cutadapt (v.3.4) and reads mapping to the human genome (GRCh37/h19—BWA v.0.7.31) were removed. The remaining reads were analyzed using Qiime2 (v.2020.8) software package (38), and a quality score filter was applied (phred score >10). Next, samples were denoised with deblur (39) and amplicon sequence variants (ASVs) were evaluated against the SILVA (v.132) database for taxonomic classification (40). Further analyses were performed using R package phyloseq (v.1.36.0) (41) and results were plotted with ggplot2 (v.3.3.5) (36). ASVs represented by less than 3 reads were discarded and as most samples almost reached saturation with 1,750 reads; only those above this limit were considered for further analysis.

Alpha (observed, Chao1, Simpson, and Shannon) and beta (Bray–Curtis, unweighted and weighted Unifrac) diversity analyses were performed utilizing R package phyloseq (v.1.36.0). Additionally, non-parametric tests were used to evaluate the differential abundances of alpha and permutational analysis of variance (PERMANOVA/ADONIS), using 999 permutations, to calculate the significance of differences in beta diversity indexes (R package vegan, v2.5-7). The linear discriminant analysis effect size (LEfSe) was used to evaluate bacterial differential abundances among samples (42), and phylogenetic investigation of communities by the reconstruction of unobserved states (PICRUSt) was used to predict the functional composition of a metagenome using the reads from 16S rRNA gene sequencing (43). To compare the differences in phyla and genera abundance between groups, raw counts were normalized by dividing the number of reads obtained for each taxon by the total number of reads from that sample.

### 2.5 Statistical Analyses

Clinicopathological and lifestyle variables were collected through medical records and questionnaires. Fisher’s exact test and chi-square tests were used for qualitative variables and Wilcoxon and Mann–Whitney U tests for quantitative variables, when appropriate (Statistical Package for the Social Sciences (SPSS), IBM v.17.0; Chicago, IL, United States). The comparisons between clinicopathological, lifestyle variables, microbiota composition, and mutations in rectal cancer were performed with Fisher’s exact test and *p-*values <0.05 were considered to be statistically significant.

## 3 Results

### 3.1 Patients’ Characteristics

At the ACCCC, a total of 41 LARC patients were recruited, and after selection and pairing, a total of 26 LARC patients (R = 11; NR = 15) were included for Brazil. The cohort from Argentina consisted of 18 patients (R = 6; NR = 12). The clinicopathological and lifestyle features of the 44 LARC patients are summarized in **Table 1**. The BR cohort was treated exclusively with nCRT, while the AR cohort had eight patients treated with nCRT and ten with TNT. Both groups showed no statistical differences in terms of age, gender, tumor location, or staging, as well as the clinical variables and pCR analysis. As clinical characteristics were homogeneous between the patients from the two countries, they were combined in a larger cohort for further analysis. The median age at diagnosis was 58 years; most patients were men (62.4%), with tumors of T3 (65.9%) and N1 (68.2%) stages; 77.3% were treated with nCRT and 22.7% with TNT. A significant association was observed between perineural invasion and poor response to treatment (*p-*value = 0.007), while no other significant associations were observed in the analysis of clinical characteristics and pathological response between both treatment regimens.

**Table 1:** Clinicopathological and lifestyle characteristics of LARC patients from Argentina and Brazil enrolled in this study.

Characteristics	Number of patients (n = 44)	Response to nCRT		*p-value*
		NR (n = 27)	R (n = 17)	
**Median age at diagnosis**	58 (34–79)	58 (34–79)	63 (43–77)	
**Country**				
Argentina	18 (40.9%)	12 (44.4%)	6 (35.3%)	0.775^b^
Brazil	26 (59.1%)	15 (34.1%)	11 (64.7%)
**Gender**				
Male	27 (62.4%)	17 (63.0%)	10 (58.8%)	1.0^b^
Female	17 (38.6%)	10 (37.0%)	7 (41.2%)
**Tumor location**				
Mid rectum	20 (45.5%)	14 (51.8%)	6 (35.3%)	0.445^b^
Low rectum	24 (54.5%)	13 (48.2%)	11 (64.7%)
**T stage pre-treatment**				
T2	5 (11.4%)	2 (7.4%)	3 (17.7%)	0.587^a^
T3	29 (65.9%)	19 (70.4%)	10 (58.8%)
T4	10 (22.7%)	6 (22.2%)	4 (23.5%)
**N stage pre-treatment**				
N0	12 (27.3%)	5 (18.5%)	7 (41.2%)	0.176^a^
N1	30 (68.2%)	21 (77.8%)	9 (52.9%)
N2	2 (4.5%)	1 (3.7%)	1 (5.9%)
**CEA pre-treatment**				
≤5	24 (54.5%)	13 (48.2%)	11 (64.7%)	0.617^b^
≥5	20 (45.5%)	14 (51.8%)	6 (35.3%)
**Alcohol consumption**				0.559^b^
No	25 (56.8%)	14 (51.8%)	11 (64.7%)
Yes	19 (43.2%)	13 (48.2%)	6 (35.3%)
**Tobacco consumption**				0.916^a^
No	22 (50.0%)	14 (51.9%)	8 (47.1%)
Yes	13 (29.5%)	8 (29.6%)	5 (29.4%)
Former	9 (20.5%)	5 (18.5%)	4 (23.5%)	
**Neoadjuvant treatment**				0.465^a^
TNT	10 (22.7%)	7 (25.9%)	3 (17.6%)
nCRT (5-FU and capecitabine)	34 (77.3%)	20 (74.1%)	14 (82.4%)
**Mucinous differentiation**				1.0^a^
Present (>50% of tumor cells)	1 (2.3%)	1 (100%)	0 (0%)
Absent	25 (56.8%)	14 (51.8%)	11 (48.2%)
NA	18 (40.9%)	12 (44.4%)	6 (35.3%)
**Lymphovascular invasion**				0.074^a^
Present	7 (15.9%)	7 (100%)	0 (0%)
Absent	33 (75.0%)	20 (60.6%)	13 (39.4%)
NA	4 (9.1%)	0 (0%)	4 (100%)
**Perineural invasion**				0.007^a^*
Present	11 (25.0%)	11 (100%)	0 (0%)
Absent	28 (63.6%)	15 (53.6%)	13 (46.4%)
NA	5 (11.4%)	1 (20.0%)	4 (80.0%)
**Tumor budding**				0.680^a^
Present	12 (27.3%)	6 (50.0%)	6 (50.0%)
Absent	12 (27.3%)	8 (66.6%)	4 (33.4%)
NA	20 (45.4%)	13 (65.0%)	7 (35.0%)

a Fisher’s exact test.

b chi-square with continuity correction.

^*^ p-value statistically significant (p < 0.05); CEA, carcinoembryonic antigen; NA, data not available.

### 3.2 Whole Exome Sequencing Analyses

WES was performed for all 44 samples using DNA extracted from tumor biopsies collected at diagnosis, with a mean of 46 million reads/sample. On average, 94% and 83% of exonic regions were respectively covered with more than 10 or 20 reads. A total of 4,054 variants were identified (94 variants/sample), including: 93 frameshift deletions, 37 frameshift insertions, 34 in-frame deletions, 6 in-frame insertions, 3,567 missense mutations, 195 nonsense mutations, 4 nonstop, and 118 splice site. A single sample derived from an AR NR patient was classified as hypermutated as it presented a missense mutation in exon 19 of the *MLH1* gene, leading to 1,511 variants just in this particular tumor sample, a significantly higher number as compared to non-hypermutated tumors, which presented a median of 59 somatic variants. A further investigation of this patient’s white blood cell DNA confirmed the presence of the same mutation in his germline lineage, suggesting a Lynch syndrome diagnosis, and some of the patient’s relatives were contacted to receive genetic counseling. We also analyzed AR and BR samples separately, and both cohorts presented a similar mutational pattern, characterized by the predominance of SNV variants, classified as missense, mostly C>T transitions (**Supplementary Figures S1**, **S2**).

**Figure 1:**
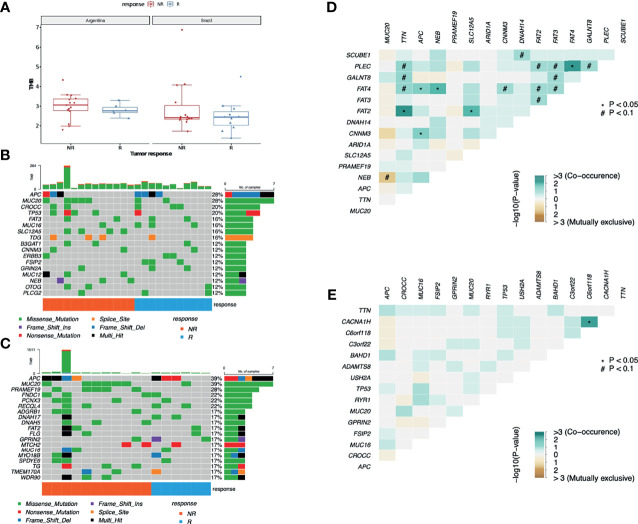
Mutational profile from LARC samples. **(A)** TMB comparison between AR and BR pre-treatment biopsy samples from R and NR patients diagnosed with LARC (Wilcoxon test *p-*value = 0.4 for AR R *vs.* NR; *p*-value = 0.58 for BR R *vs.* NR). **(B)** Distribution of somatic mutations found in pre-treatment biopsies of LARC in BR and **(C)** AR samples. Each column represents a patient, and each line represents a gene. The upper plot shows the number of mutations (TMB) in each sample, the central plot shows the mutation types as indicated by the colors, the right plot indicates the number of samples with mutations in that specific gene, and the lower part of the figure indicates the response of each patient (R, responder; NR, non-responder). **(D)** Co-occurrence of genetic alteration analysis in LARC biopsies before neoadjuvant treatment obtained from NR and **(E)** R patients.

We have also investigated the association of TMB and response to nCRT, yet no statistical significance was found (Mann–Whitney test, *p-*value = 0.1096). When the samples from each country were assessed separately, considering the response to the therapy used, although samples from Argentina presented a higher TMB, no significant differences were observed between R and NR cohorts (Mann–Whitney test, *p-*value AR samples = 0.2225; *p-*value BR samples = 0.2543) (**Figure 1A**). ITH was inferred using the mutant allele tumor heterogeneity (MATH) score (maftools R package), where the tumors with a higher number of distinct cellular populations present greater scores. The WES-MATH scores obtained from LARC samples ranged from 14.7 to 73.2 (median = 39.4, mean = 40.9). However, we observed no associations between the MATH values and the treatment response (*p-*value = 0.3524, Mann–Whitney test), country of origin (*p-*value = 0.3173, Mann–Whitney test), T-stage (*p-*value = 0.3789, Mann–Whitney test), or N-stage (*p-*value = 0.0854, Mann–Whitney test).

Leaving aside the FLAGS genes (44), the most mutated genes were *APC* and *MUC20*, both with mutations in 28% of the BR cohort and in 39% of the AR cohort, followed by *TP53* (altered in 11% of AR samples and 20% of the BR ones), and *CROCC* (mutations in 6% of AR patients and in 20% of the BR patients). Also, tumors presented an important interindividual heterogeneity and identical mutations among the distinct patients in both cohorts were rare (**Figures 1B, C**). Interestingly, despite being a FLAGS gene, *MUC16* mutations were detected in 24% of R and 12% of NR patients, although the statistical significance level was not reached. Furthermore, neither the mean number of mutations nor any of the top 30 mutated genes were distinct between the groups with different responses to nCRT treatment. At last, the analysis of mutual exclusivity and co-occurrence of mutations suggests that concurrent mutations in tumor suppressor genes *APC* and *FAT4* are significantly correlated with the lack of response to nCRT (*p-*value < 0.05) (**Figures 1D, E**).

**Figure 2:**
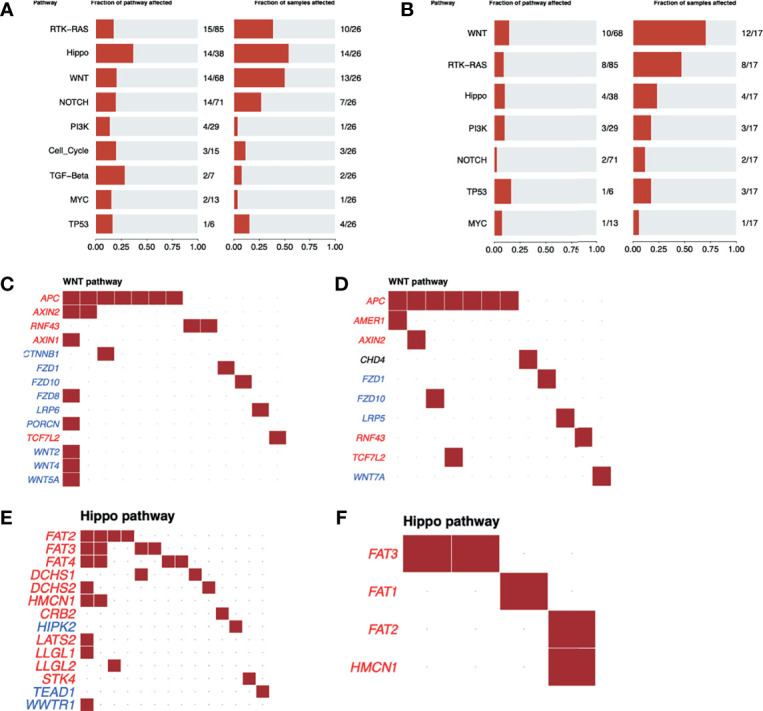
Oncogenic pathway analysis in WES data from LARC. The most altered oncogenic pathways in LARC biopsies before neoadjuvant treatment in **(A)** NR and **(B)** R patients. Wnt-β catenin oncogenic pathway alterations in LARC biopsies before neoadjuvant treatment in **(C)** NR and **(D)** R patients. Tumor suppressor genes are represented in red and oncogenes in blue. Each square represents a sample with a mutation in the respective gene. Hippo oncogenic pathway alterations in LARC biopsies before neoadjuvant treatment in **(E)** NR and **(F)** R patients. Tumor suppressor genes are represented in red and oncogenes in blue. Each square represents a sample with a mutation in the respective gene.

Although the Wnt-β catenin pathway was the most frequently altered in R (70% *vs.* 50% in NR) (**Figure 2A**), the Hippo pathway was the most affected in NR (54% compared to 23.5% in R) (**Figure 2B**). In addition, while the Wnt-β catenin pathway presented a similar mutational profile between groups with distinct responses to nCRT (**Figures 2C, D** **)**, different genes from the Hippo pathway were observed as mutated in R and NR patients (**Figures 2E, F** **)**, with NR samples presenting 14 altered genes with at least one variant, while in the R group, only 4 genes were mutated, each with only one variant.

​​As the clusters of somatic mutations in human cancers usually present characteristics imprinted in the genome, we also investigated the putative association of mutational signatures with distinct treatment responses to the treatment therapies used (37, 45). For most patients, similar mutational profiles were found, and for this reason, we have reanalyzed our data, focusing on mutational signatures previously correlated to colorectal cancer. From this analysis, we identified the age-related signature (SBS1), signatures of unknown etiology (SBS5, SBS94, SBS89, SBS17a, and SBS17b), and just one patient exhibited a distinct mutational profile with a significant proportion of the defective DNA MMR-related signatures SBS15 and SBS26 (as previously identified by the *MLH1* gene mutation). We observed no clustering of samples regarding any clinical pathological characteristics or, for that matter, any other of the evaluated features (**Supplementary Figure S3A**). Noteworthy, NR patients presented an enrichment of the SBS5 signature when compared to R patients (*p-*value = 0.0021), yet its underlying mechanisms are not fully understood (46) (**Supplementary Figure S3B**).

### 3.3 The Rectal Tissue-Associated Tumor Microbiota

#### 3.3.1 Sequence Analyses

The V3–V4 regions from the 16S rRNA gene were successfully amplified in all 44 biopsy samples, leading to an average of 24,819 quality-filtered reads per sample. All samples reached saturation with about 1,750 reads (**Supplementary Figure S4**). A total of 2,097 ASVs were classified according to the SILVA database, and after removing sequences with less than 3 reads, 1,858 ASVs remained.

#### 3.3.2 Alpha and Beta Diversity

**Figure 3:**
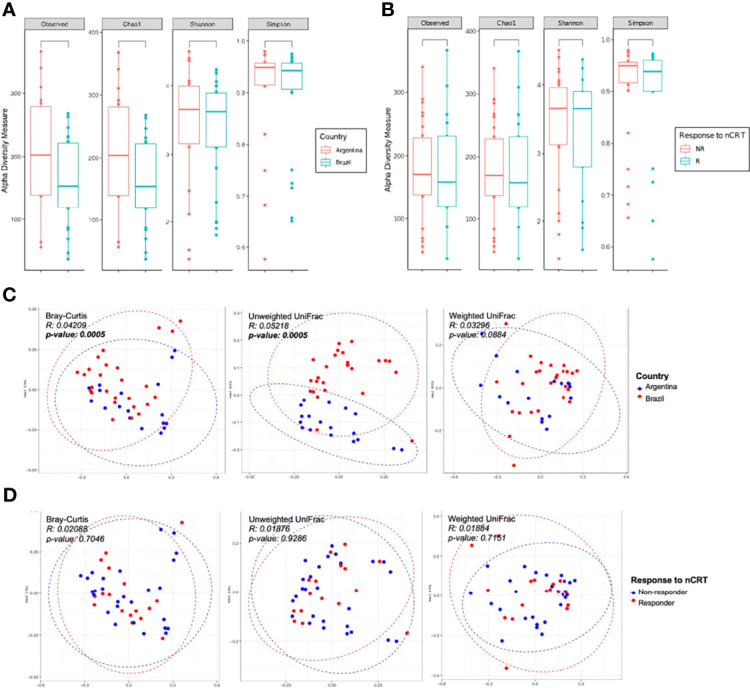
Alpha and beta diversity of LARC biopsies before neoadjuvant treatment. Boxplots showing the bacterial alpha diversity using different metrics (observed ASVs, Chao1, Shannon, and Simpson indices) between **(A)** country of origin of the samples: Argentina and Brazil; **(B)** response to neoadjuvant treatment: R and NR patients. No statistically significant differences were observed (Mann–Whitney U test, *p-*value > 0.05); PCoA ordination plots showing the bacterial beta diversity using three distance metrics (Bray–Curtis, unweighted and weighted UniFrac) comparing **(C)** country of origin of the samples: Argentina and Brazil; and **(D)** response to neoadjuvant treatment: R and NR patients. Samples from Argentina and Brazil formed two separate clusters (Bray–Curtis and unweighted UniFrac distances, PERMANOVA/ADONIS *p-*value < 0.05).

The LARC-associated microbiota in AR tissue samples presented a non-significant trend toward an increased number of observed ASVs, as well for increased richness (Chao 1 estimator), as compared to the BR tissue samples (*p*-values = 0.07 and 0.068, respectively) (**Figure 3A**). When patients were stratified as R and NR, we observed no significant differences in any of the evaluated alpha indexes (observed, Chao1, Shannon, and Simpson, *p*-value > 0.05) (**Figure 3B**). We also evaluated species richness between low and medium rectum samples (**Supplementary Figure S5**), and CAP 0 samples *vs*. other regression grades (**Supplementary Figure S6**), yet similar microbial diversity were again observed from these analyses (observed, Chao1, Shannon, and Simpson, *p*-value > 0.05).

The comparison between AR and BR samples showed statistically significant differences in its bacterial composition (Bray–Curtis and unweighted Unifrac, *p-*value = 0.005, ADONIS using 999 permutations) (**Figure 3C**). However, similar abundance and phylogenetic distances were observed between R and NR patients (**Figure 3D**), as well as in low and medium rectum samples (**Supplementary Figure S7**) and CAP 0 samples *vs.* other regression grades (**Supplementary Figure S8**) (Bray–Curtis, unweighted Unifrac, and weighted Unifrac, *p-*value > 0.05, ADONIS using 999 permutations).

#### 3.3.3 Microbial Communities

A total of 16 phyla, 25 classes, 48 orders, 76 families, and 219 genera were identified in all samples. Moreover, at least 1.72%, 1.76%, and 5.7% of ASVs were respectively classified as uncultured, uncultured bacterium or NA at the genus level. These ASVs were considered individually in our analysis.

**Figure 4:**
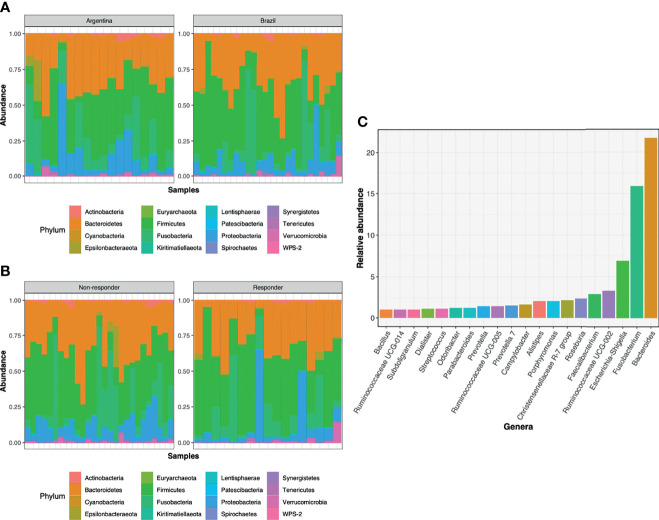
Main bacterial phyla and genera in pre-treatment biopsies of LARC: **(A)** Relative abundance of the main phyla according to the **(A)** country of origin of the samples and **(B)** response to neoadjuvant treatment: R and NR patients. **(C)** Relative abundance of bacterial genera from Argentina and Brazil, with relative abundance above 1%.

At the phylum level, bacterial composition of AR and BR samples concerning treatment responses was similar, with three phyla contributing with more than 85% of the microbiota. Bacteroidetes was the most predominant phylum in BR biopsies (36.2% v*s.* 31.2% in AR) and Firmicutes in AR samples (32.4% *vs.* 36.2% in BR) (**Figure 4A**). When the patients from both countries were combined and then stratified in R and NR, Bacteroidetes was more abundant in NR (35.2% *vs.* 31.6% in R), while in R patients’ biopsies, the most dominant phylum was Firmicutes (38.0% *vs.* 31.7% in NR, respectively) (**Figure 4B**), and no significant differences were observed between the samples from different countries or cohorts with different responses to treatment (Wilcoxon-test, *p-*value > 0.05).

From all the genera identified, only 38% and 10% presented relative abundances of above 0.1% or 1%, respectively (**Figure 4C**). Considering the 10 most abundant genera associated with the rectal mucosa, similar profiles were observed between AR and BR, as well as in the groups with a distinct response to nCRT (**Table 2**).

**Table 2:** Top 10 genera identified in pre-treatment LARC biopsies according to the country of origin and patient’s response to neoadjuvant treatment (nCRT).

Top 10	NR patients		R patients	
	Genus	Frequency (%)	Genus	Frequency (%)
**Brazil**				
1	*Bacteroides*	28.9	*Bacteroides*	25.8
2	*Fusobacterium*	7.1	*Fusobacterium*	21.1
3	*Faecalibacterium*	5.3	*Escherichia-Shigella*	4.1
4	*Roseburia*	5.0	*Roseburia*	3.4
5	*Escherichia-Shigella*	4.8	*Faecalibacterium*	3.2
6	*Ruminococcaceae UCG-002*	4.4	*Dialister*	2.6
7	*Alistipes*	2.8	*Alistipes*	2.4
8	*Christensenellaceae* R-7 group	2.5	*Streptococcus*	2.2
9	*Odoribacter*	2.4	*Prevotella*	2.2
10	*Porphyromonas*	1.5	*Bacillus*	2.0
**Argentina**				
1	*Fusobacterium*	21.6	*Escherichia-Shigella*	13.3
2	*Bacteroides*	20.0	*Fusobacterium*	12.7
3	*Escherichia-Shigella*	6.4	*Bacteroides*	10.2
4	*Campylobacter*	3.7	*Ruminococcaceae UCG-002*	5.8
5	*Prevotella 7*	3.0	*Christensenellaceae R-7 group*	4.2
6	*Porphyromonas*	2.9	*Rikenellaceae RC9 gut group*	3.4
7	*Ruminococcaceae UCG-002*	2.5	*Porphyromonas*	3.2
8	*Christensenellaceae R-7 group*	1.8	*Prevotella*	2.5
9	*Peptostreptococcus*	1.8	*Ruminococcaceae UCG-005*	2.4
10	*Acinetobacter*	1.7	*Faecalibacterium*	2.3

**Figure 5:**
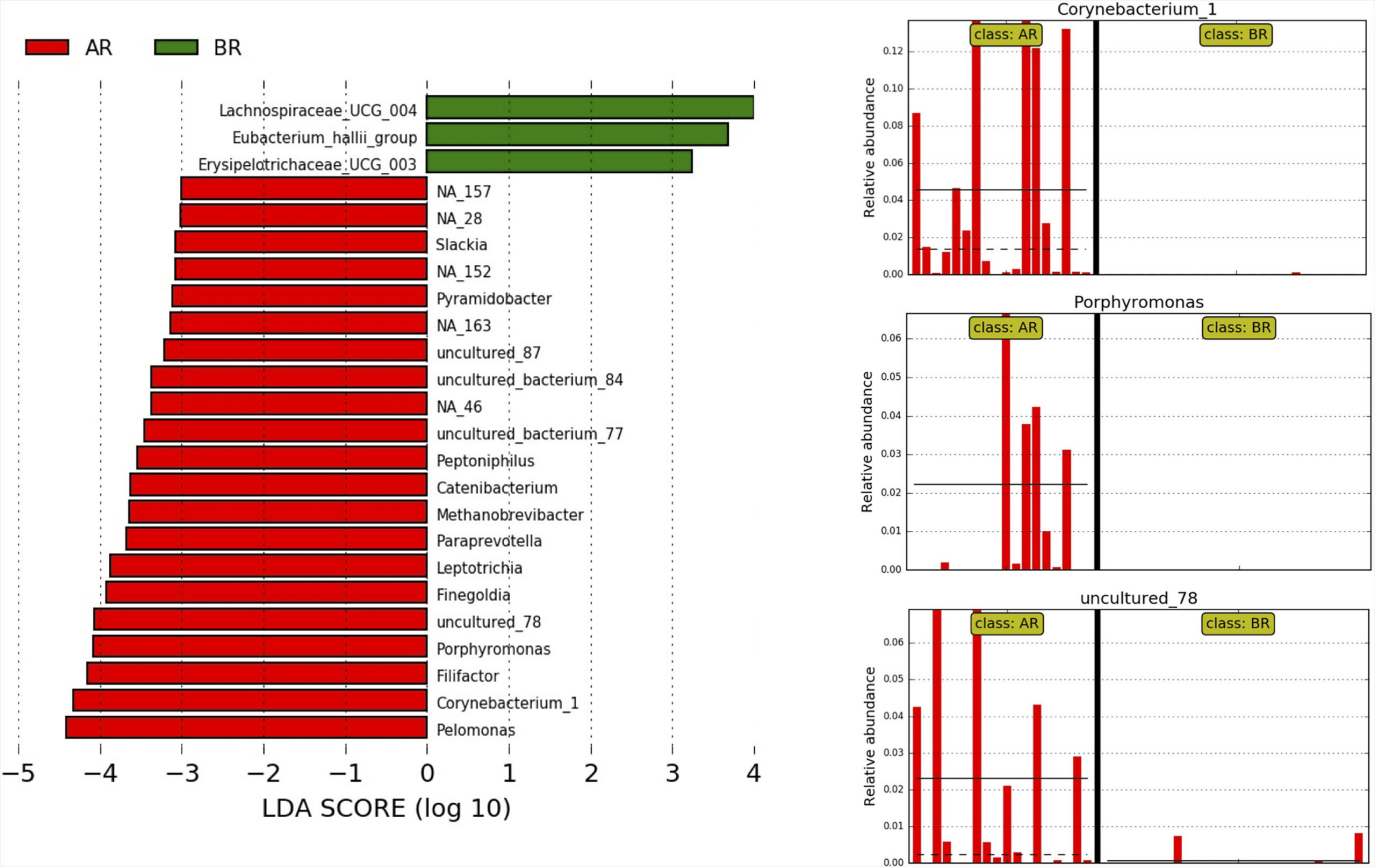
LEfSe at the genus level for pre-treatment LARC biopsies according to the country of origin. AR samples are indicated by red and BR samples by green; horizontal bars represent the effect size for each genus, and the bar length represents the log10 LDA score, indicated by the dotted lines (vertical). The three plots on the right highlight the genera present almost exclusively in AR samples.

A high-dimensional analysis comparing the cohorts from both countries by the LEfSe identified 24 genera differentially enriched between the samples from Argentina and Brazil (LDA score ≥3), five of them with an LDA score ≥4. The genus *Corynebacterium_1* (mean relative abundance of 0.28% in AR and 0.02% in BR samples), *Porphyromonas* (mean relative abundance of 1.7% in AR and 0.16% in BR samples) and uncultured_77 (mean relative abundance of 0.11% AR and 0.04% in BR samples) were all more abundant in the samples from Argentina (**Figure 5**).

**Figure 6:**
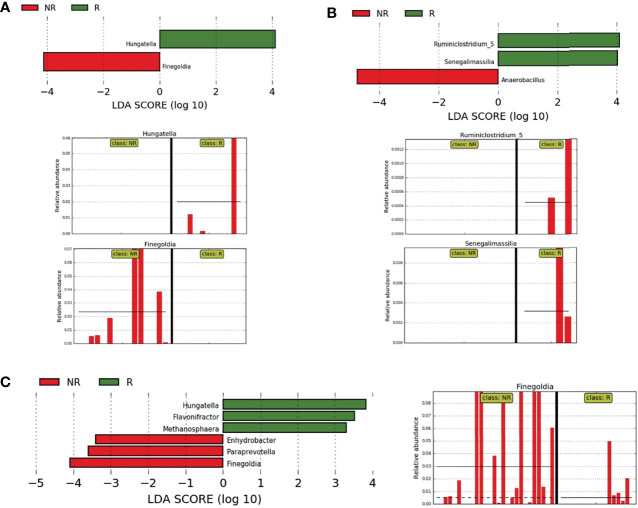
Differently abundant bacteria between R and NR. **(A, B)** LEfSe at the genus level for pre-treatment LARC biopsies according to the country of origin and response to neoadjuvant treatment. NR samples are indicated by red and R samples by green; horizontal bars represent the effect size for each genus and bar length represents the log10 LDA score, indicated by the dotted lines (vertical). **(A)** BR R and NR patients, the two plots in the bottom highlights genera present exclusively in NR and R samples; **(B)** AR R and NR patients, the two plots in the bottom highlight genera present exclusively in R samples. **(C)** LEfSe at the genus level for pre-treatment LARC biopsies from Argentina and Brazil according to the response to neoadjuvant treatment. NR samples are indicated by red and R samples by green; horizontal bars represent the effect size for each genus and bar length represents the log10 LDA score, indicated by the dotted lines (vertical). The plot in the bottom highlights the genus *Finegoldia* present almost exclusively in NR samples.

When the samples from each country were evaluated separately, comparing R and NR groups, the genus *Hungatella* was identified exclusively in R patients, while *Finegoldia* was found only in NR, both from the BR cohort (LDA score ≥4) (**Figure 6A**). On the other hand, in the samples from Argentina, the genera *Ruminiclostridium_*5 and *Senegalimassilia* were identified only in R, while in NR, we observed a higher abundance of *Anaerobacillus* (LDA ≥ 4) (**Figure 6B**).

When combining samples from both countries, three genera were enriched in R samples (LDA ≥3): *Flavonifractor* (mean relative abundance of 0.13% in R *vs.* 0.03% in NR)*, Hungatella* (mean relative abundance of 0.57% in R *vs.* 0.07% in NR) and *Methanosphaera* (mean relative abundance of 0.02% in R and absent in NR). On the other hand, *Enhydrobacter* was exclusively present in NR samples (LDA ≥ 3, mean relative abundance of 0.10%), while *Paraprevotella* and *Finegoldia* were enriched in NR samples, the last one with LDA ≥ 4 (**Figure 6C**).

**Figure 7:**
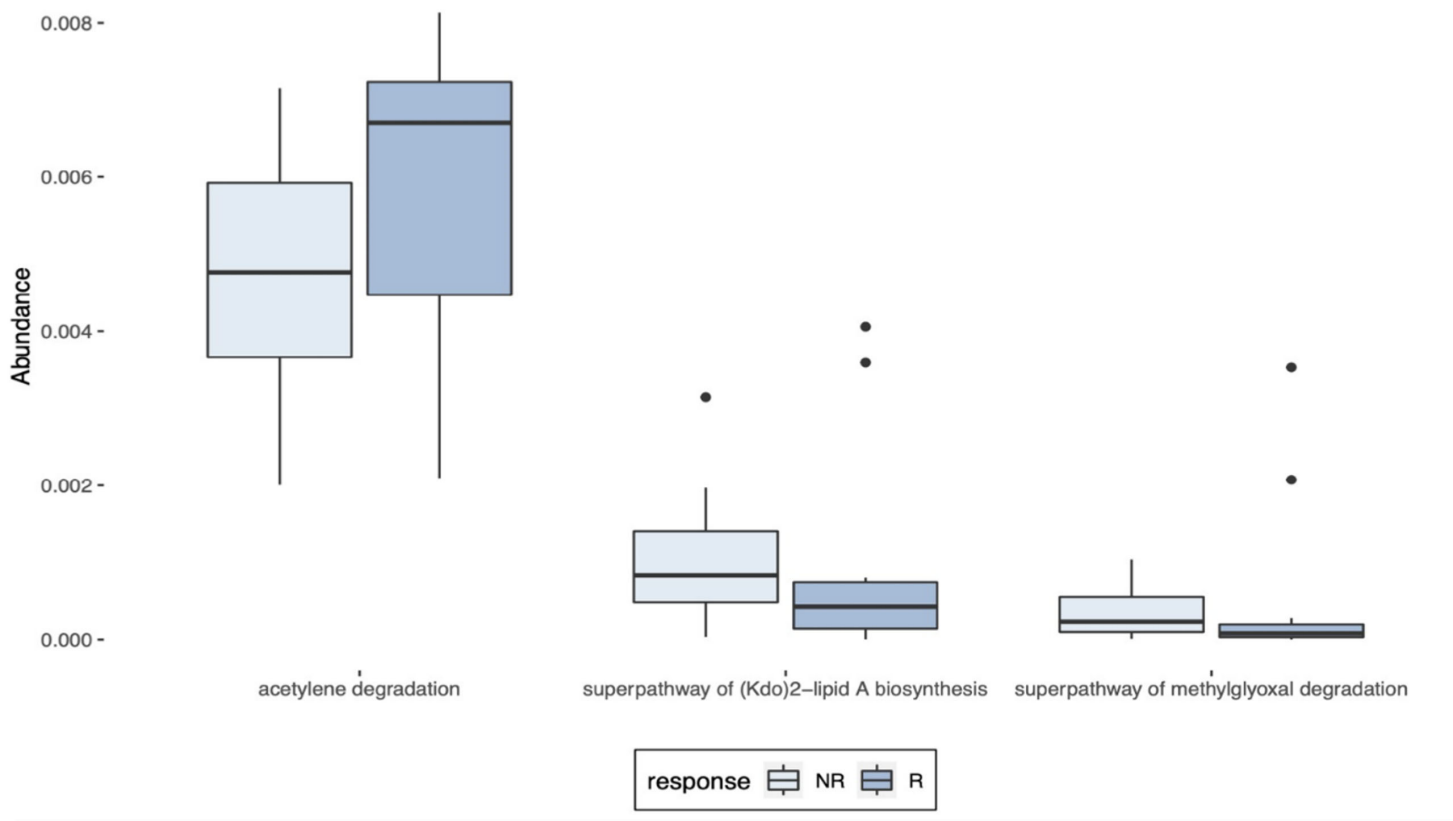
Predictions of the metagenomes identified in LARC biopsy samples. PICRUSt predictions of the metagenomes previously identified as differentially abundant in pre-treatment LARC biopsies between R and NR patients as identified by LEfSe (LDA score ≥ 2).

PICRUSt was used to indicate the function and pathways of the metagenomes previously identified as differentially abundant between R and NR patients by the LEfSe, the LDA score ≥2. Increased acetylene degradation was observed in R, while higher Kdo2-lipi A biosynthesis and methylglyoxal degradation were detected in NR biopsies (**Figure 7**).

## 4 Discussion

The management of LARC patients has changed over the years, and although better survival rates were reached with multimodality treatment approaches, the achievement of complete pathological response rates is still occasional. In our cohorts, 16.7% of AR and 27% of BR patients reached pCR to nCRT, values within the 10%–30% range described in the literature (1) with similar rates between nCRT and TNT-treated patients. Diverse clinical, radiological, pathological, and molecular factors have been associated to LARC treatment efficacy. Whereas all these contribute to the understanding of the biology of therapeutic response, we still have no markers reaching the precision required for clinical applications, when responsive patients identified prior to cancer treatment would benefit from the adoption of non-operative therapies (“watch and wait”) (3, 47) and refractory subjects could be spared from the conventional nCRT treatment. Despite the continuous search for histological, serological, cellular, and molecular markers, there are no established predictive factors to the response to nCRT in rectal cancer (48). In the absence of reliable markers, most patients worldwide are blindly subjected to the standard neoadjuvant chemotherapy treatment regimens, the current gold standards to LARC care. Here, we studied the mutation profiles determined by WES analysis and evaluated the tumor tissue-associated microbiota collected at diagnosis, as tools to investigate the potential markers of pCR.

The previous WES studies from The Cancer Genome Atlas-Rectum adenocarcinoma (TCGA-READ) (49), after discarding the FLAGS genes *TTN* and *MUC16*, indicate the following top five most frequently mutated genes: *APC* (88.3%), *TP53* (78.1%), *KRAS* (40.9%), *FAT4* (21.2%), and *FBXW7* (17.5%). For the BR cohort, the WES-derived mutation rates for these same five genes were as follows: *APC* (28%), *TP53* (20%), *KRAS* (0%), *FAT4* (8%), and *FBXW7* (4%), while in the AR cohort, they were as follows: *APC* (39%), *TP53* (11%), *KRAS* (6%), *FAT4* (11%), and *FBXW7* (6%). As our low vertical coverage (54×) and variable horizontal coverage among genes and samples, associated with our requirements of allelic frequency to call the variants may lead to a false perception of a low mutation frequency, we took *KRAS* as an example to manually investigate variants in the most frequent mutations hotspots of this gene: codon 12, exon 2; codon 13, exon 2; codon 61, exon 3; and codon 146, exon 4. When cases were evaluated manually, one by one, considering only the coordinates covered by at least 5 reads, we saw 45% mutation rate (42% in BR and 50% in ARG samples), reinforcing that our coverage is likely to reveal just the most frequent mutations in our cohort. As we could not ensure the veracity of these mutations, we opted to be conservative and report only the variants called using our stringent filters. The other elements to be considered include the limited number of patients enrolled in our study, the heterogeneity of the tumor biopsies the variable percentage of tumor cells, and the lack of a matched non-tumor tissue filter, among others. Nevertheless, Ye et al. (2018) (50) also reported that the exome sequencing of Chinese LARC patients (average coverage depth 99.3×) showed a much lower somatic mutation distribution compared to the TCGA genes: *APC* (36% *vs*. 62%) and *TP53* (28% *vs*. 57%), in accordance with other Chinese studies. Our WES analysis did not point genetic variants that allowed to segregate response to treatment, such as candidate variants previously reported as associated with pCR by Lee et al. (18).

Although being a FLAGS gene, *MUC16* mutations were previously associated with improved prognosis, as it enhances the antitumor immune responses through cytotoxic T lymphocytes in endometrial (51) and gastric cancers (52) and was associated with better response and treatment outcomes after therapy with immune checkpoint inhibitors (53). A finding of potential interest was the increased mutation rate found in *MUC16*, which was detected in 24% of R and 12% of NR patients (Fisher’s exact test, *p-*value = 0.4069).

Yang et al. (2019) (54) identified three mutational signatures in pre-treatment LARC samples: one that did not resemble any COSMIC signatures, another that seemed to be a combination of more than one COSMIC signature, and SBS1, an age-related signature. In accordance with this study, our samples presented not only the SBS1 signature but also SBS5, this being enriched in NR samples (Mann–Whitney test, *p-*value = 0.0021). Although its etiology is unknown, SBS5 is known as a “clock-like” signature, as the number of mutations increases with an individual’s age. It is also associated with tobacco smoking, although no differences in tobacco consumption were observed between R and NR (*p-*value = 0.916) (https://cancer.sanger.ac.uk/signatures/sbs/sbs5/).

Additionally, a noteworthy finding of this study is the identification of *APC* and *FAT4* co-occurrence mutations exclusively in NR patients (*p-*value < 0.05). *FAT4* is a conserved member of the cadherin superfamily, which is involved in cell-to-cell adhesion (55), capable of suppressing tumor growth through Hippo signaling activation (56), as well as activating the Wnt-β catenin signaling (56). It was found recurrently mutated in melanoma, pancreatic, breast (57), and gastric cancers (58). In colorectal tumors, *FAT4* was described as a novel recurrently mutated gene (prevalence of 14%) (59), and more recently, it was also implicated in the regulation of the PI3K/AKT signaling pathway, inhibiting the epithelial-to-mesenchymal transition in CRC cells (60). Although *FAT4* mutations have been described in colorectal cancers before, their role in response prediction is still unknown.

It was reported that PDAC patients treated with gemcitabine and harboring deletions or inactivating mutations in Hippo pathways presented shorter survival due to drug resistance (61). As the highly mutated Wnt-β catenin pathway is in part regulated by Hippo (62) and mutations that potentially inhibit the Hippo pathway were more prevalent in the NR group, the concurrent mutations in *APC* and *FAT4* could be potential markers of treatment resistance. In the same line, Sendoya et al. (2020) and Kamran et al. (2019) (11, 63) similarly reported that simultaneous *RAS* and *TP53* mutations in LARC patients with a proficient DNA repair system were associated with poor responses to nCRT.

The microbiota associated to the rectal tumor tissues was also distinct between AR and BR tumors. AR samples had a trend toward a higher number of ASVs, as well as an increase in richness as measured by Chao1. Also, when response to treatment was not considered, the beta diversity between the LARC-associated microbiota were significantly distinct in Bray–Curtis and unweighted UniFrac distance metrics, a result expected, since geography, ethnicity, dietary factors, and tumor mutational profiles, along with other factors, may influence the gut microbiome composition (64). In this regard, the crosstalk between tissue mutational profiles in colorectal cancer and bacteria associated to these tumors has been well described in patients with Lynch syndrome and familial adenomatous polyposis (65, 66), as well as in sporadic CRC (67). The genetic mutation profiles characteristic from CRC appear to shape the tumor-associated microbiota, and the combination of a set of bacteria was able to predict the loss of function of specific genes, such as *APC* and *ANKRD36C* (63). Furthermore, the tumor-associated microbiota could be correlated with the consensus molecular subtypes of CRC (68).

The relevance of the tumor-associated microbiota is increasingly being recognized in the literature (25, 69) not only as a surrogate to cancer detection (20, 70) but also as an agent that is capable to interfere with the cancer therapy (21) and survival, as demonstrated in patients with resected PDAC (24). A previous work by our group showed the dysbiosis observed in rectal tumor tissues, including a substantial increase of species richness and diversity in the tumor as compared to non-tumor tissue samples (20). In colorectal cancer, *F. nucleatum* secretes adhesion and virulence factors that modulate the microenvironment, maintaining a proinflammatory state that potentiates carcinogenesis (71). Specifically for rectal tumors, an increased abundance of *Fusobacteria* was observed in intermediate and poor responders to nCRT (29), and although baseline *F. nucleatum* levels were not associated with response, its positivity after nCRT significantly increased the risk of tumor relapse (26).

In our study, we found three bacteria genera by the LEfSe analysis to be increased in nCRT responders in both AR and BR cohorts: *Hungatella*, *Flavonifractor*, and *Methanosphaera*, all of them presenting LDA scores ≥3). Taylor (2021) (72) analyzed the microbial transcription and hypothesized that *Hungatella hathewayi, F. nucleatum, Butyricimonas faecalis, Alistipes finegoldii, Bacteroides thetaiotaomicron*, and *B. fragilis* may contribute to tumor regression by modulating both the metabolism and the immune responses, which could explain our findings of increased levels of *Hungatella* and *Fusobacterium* in R individuals. Fecal microbiota studies from LARC patients treated with nCRT showed *Hungatella* to be associated with less toxicity to treatment (73). Others described that the *Flavonifractor* genus is a butyrate producer, a short-chain fatty acid (SCFA) related to colon health, that stimulates the production of mucin and is enriched in the Tunapuko hunter-gathered individuals (74). Furthermore, the species *Flavonifractor plautii* appears to be one of the few gut bacteria capable of biotransforming quercetin, an anti-inflammatory flavonoid with preventive roles in CRC, into its biologically active form (75). At last, *Methanosphaera* is an indigenous gut microbiome Archaea, especially *Methanosphaera smithii*, which is the most abundant species known from this kingdom. The *Methanosphaera* genus was associated to pathogenic conditions but is also capable to activate innate immune cells. Both *M. smithii and M. stadtmanae* were shown to activate monocyte-derived dendritic cells (mo-DCs) and especially the late, appears to contribute to pathological conditions in the gut. The role of *M. stadtmanae* can be quite relevant, as this species was able to strongly activate *in vitro* both receptors CD86 and CD197, which are pivotal in the maturation of mo-DCs that can be further involved in adaptative immune responses (76). When all these aspects are taken together, it is reasonable to conciliate the presence of these microorganisms and a complete response phenotype.

The three genera found by LEfSe analysis to be correlated with NR to nCRT, *Enhydrobacter, Paraprevotella*, and *Finegoldia* had LDA scores above 3 (the LDA score of *Finegoldia* reached ≥4). Curiously, the species *Enhydrobacter aerosaccus (*formerly *Moraxella osloensis*) was recently described to be enriched in the cervical cancer group microbiome (77) and was also found to be associated to the adenomas in the gut (78). *Paraprevotella* was associated to CRC tissues before (78) and was also enriched in feces from CRC patients, when compared with the tumor tissue and feces from controls (79). Finally, yet importantly, *Finegoldia* was a genus with a higher abundance in oral tumors compared to controls (80). *Finegoldia magna* (formerly *Peptostreptococcus magnus*), the only species of this genus described so far, is a highly successful opportunist pathogen and also the most pestilent of the Gram-positive anaerobic cocci (81) *F. magna* has many virulence factors that facilitate the invasion of epithelia, neutralization of defenses, and a strong attachment to the tissues and production of resistant biofilms that helps in the chronification of infections, turning them into wounds (82). Actually, *Finegoldia* was found to be associated to the biofilms of three types of chronic wounds that are challenging to heal (83, 84). Besides using the biofilms to be protected from the immune response orchestrated by the host, and many times also from antibiotic treatment, *F. magna* also uses the neutrophil extracellular traps to hide from the immune system and to replicate (84). Curiously, *Finegoldia* spp. was found to be associated to colorectal adenomas but not with a normal colon (85). Even more instigating was the finding by Burns et al. (2018) (68), who observed that the CRC tumors with loss of function in the *APC* gene presented an increased abundance of *Finegoldia*, although this correlation was not found in our cohort. Ultimately, yet very intriguing, one R patient presented a relatively high abundance of *Finegoldia* in the pre-treatment biopsy, contrasting our findings at first sight. However, further investigation identified that this same individual presented disease progression in less than one year after surgery, suggesting not only the role of the *Finegoldia* genus in identifying patients who are more likely to be NR, but also as a potential marker for patients with an enhanced risk of progression.

Summing up this information, the evaluation of *Enhydrobacter, Paraprevotella*, and *Finegoldia* genera together as predictive biomarkers of response to the nCRT treatment in LARC is promising. However, the validation of these findings in larger LARC cohorts treated with nCRT, ideally including samples derived from distinct locations, with variable genetic background, diet, and lifestyle should be granted. Additionally, because our study is based on targeted sequencing, limited to genus identification, it is also important to investigate which species are associated with the putative biological role of these genera in LARC treatment.

Finally, despite the low abundances, PICRUSt analysis indicated three metabolic pathways that are significatively different between R and NR. An increase in methylglyoxal degradation was observed in NR, pointing to a higher concentration of this highly toxic metabolite produced due to the enhanced metabolic reprogramming of cancer cells. The distress induced by methylglyoxal could not only promote protein and nucleic acid glycation, but also enhance the metastatic dissemination of breast cancer cells (86). In addition, the Kdo_2_-lipid A biosynthesis was increased in NR patients, and lipid A is a strong immunoreactive endotoxic center of lipopolysaccharide (87), which, when combined to methylglyoxal, could shape the tumor microenvironment to a pro-inflammatory state, possibly explaining our findings. On the other side, in R patients, the acetylene degradation pathway was substantially enriched. As acetylene can be metabolized to acetyl-CoA and then, in acetate and butyrate, this increased production of anti-inflammatory SCFA, combined with the higher abundance of the bacterial genera producer of SCFAs, could help to understand our results (88).

Our study included 44 patients, belonging to two different cohorts, treated with the current gold standards to LARC care, and pointing to bacteria that may play a role in treatment response. Besides the relatively small sample size, we have extended the current characterization of the exome of the rectal cancer tissue and described for the first time the composition of the pre-treatment LARC tissue-associated microbiota. Although a proper validation of our findings in a larger sample size is still needed to increase the detection power, while reducing the likelihood of a type II error, our study described the co-occurrence of *APC* and *FAT4* mutations, as well as increased abundances of *Enhydrobacter, Paraprevotella*, and *Finegoldia* in LARC biopsies as potential predictive markers of response to nCRT, which may not only help to select patients more likely to respond to treatment, but may also lead to tailored approaches to improve the therapeutic response of these patients.

## Data Availability Statement

The datasets presented in this study can be found in online repositories. The names of the repository/repositories and accession number(s) can be found below: NCBI under accession number PRJNA778982 (https://dataview.ncbi.nlm.nih.gov/object/PRJNA778982?reviewer=g4tsogkjujrgdpialdg2n7f61f&archive=biosample).

## Ethics Statement

The studies involving human participants were reviewed and approved by Comitê de Ética em Pesquisa em Seres Humanos da Fundação Antônio Prudente - A.C. Camargo Cancer Center. The patients/participants provided their written informed consent to participate in this study. Written informed consent was obtained from the individual(s) for the publication of any potentially identifiable images or data included in this article.

## Author Contributions

Experimental design: IT, TB, CM, RR, ED-N, SJ, and DN. Patient recruitment and sample collection: IT, JS, MG, JR, and BK. Sample preparation and experimental procedures: IT, TB, MS, GB, LS, GO, and LC. Bulk data processing: AD and IS. Data analysis and interpretation: IT, TB, AD, ED-N, and DN. Results discussion: IT, TB, AD, JS, MG, JR, AL, CM, RR, ED-N, SI, SA, and DN. Manuscript writing: IT, TB, ED-N, and DN. All authors contributed to the article and approved the submitted manuscript.

## Funding

IT was supported by a fellowship from CAPES. This project received financing support from the Programa Nacional de Atenção ao Paciente Oncológico (PRONON – SISPAR 2500.055-167/2015-23), INCT (FAPESP #2014/50943-1, #2013/23277-8, and CNPq #465682/2014-6), and National Institute of Science and Technology in Oncogenomics and Therapeutic Innovation (INCITO) (2014/50943-1). ED-N is a scholar from Conselho Nacional de Desenvolvimento Científico e Tecnológico (CNPq). Funding for the recruitment and biobanking of the Argentinian cohort was provided by Fondation Nelia and Amadeo Barletta (FNAB), the FS-PBIT 015/13 grant from FONARSEC, National Agency for Promotion of Science and Technology, Ministry of Science, Technology and Productive Innovation, Argentina and the National Council for Scientific and Technological Research (CONICET), Argentina.

## Conflict of Interest

The authors declare that the research was conducted in the absence of any commercial or financial relationships that could be construed as a potential conflict of interest.

## Publisher’s Note

All claims expressed in this article are solely those of the authors and do not necessarily represent those of their affiliated organizations, or those of the publisher, the editors and the reviewers. Any product that may be evaluated in this article, or claim that may be made by its manufacturer, is not guaranteed or endorsed by the publisher.
