# Supplementary material for: Exome and Tissue-Associated Microbiota as Predictive Markers of Response to Neoadjuvant Treatment in Locally Advanced Rectal Cancer
Source: Front Oncol. 2022 Mar 22;12:809441. doi: 10.3389/fonc.2022.809441 (PMC8982181; doi:10.3389/fonc.2022.809441)
Supplement: Supplementary file 1 [file DataSheet_1.docx]

Supplementary Material

# Supplementary Figure S1. Somatic mutations identified by WES in Argentinian and Brazilian biopsies samples of locally advanced rectal cancer (LARC)

#
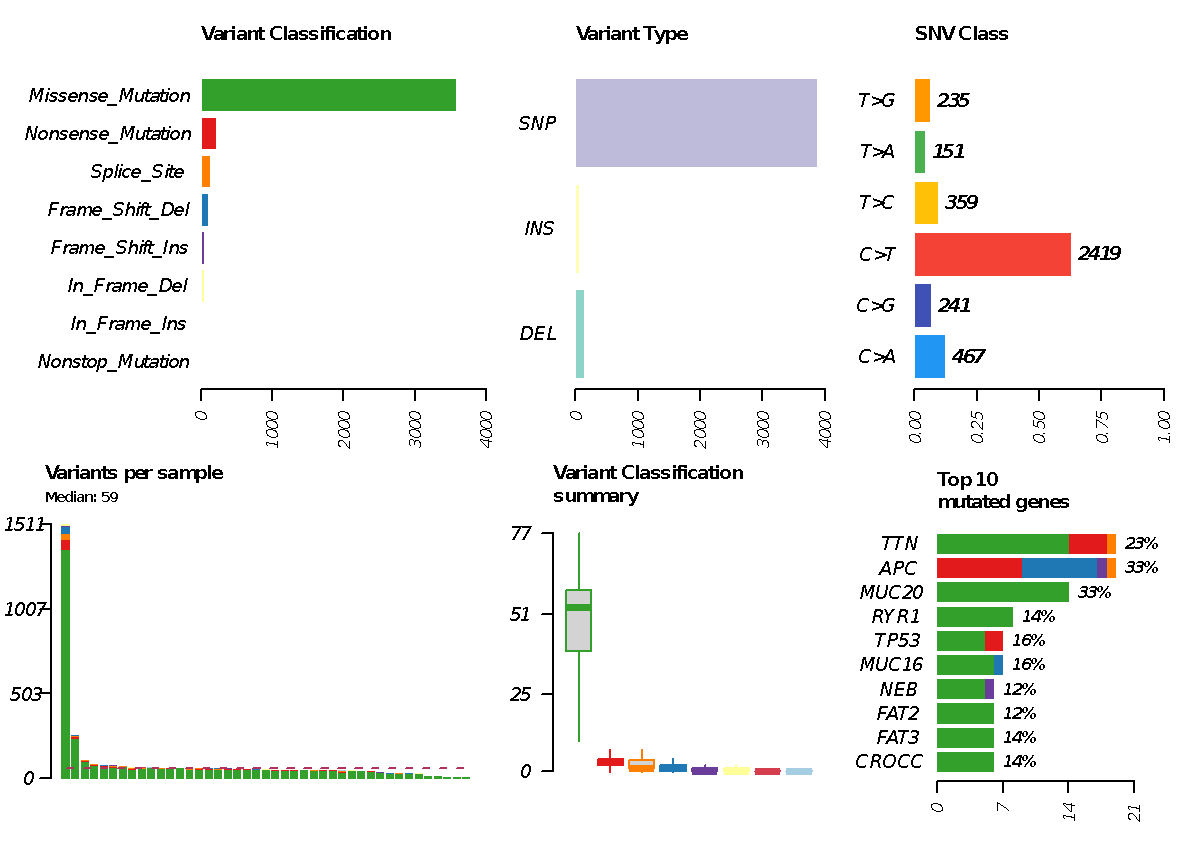


**Supplementary Figure S2.** Somatic mutations identified by WES in biopsies samples of locally advanced rectal cancer (LARC) in: **(A)** Brazilian patients; **(B)** Argentinian patients

**(A)**


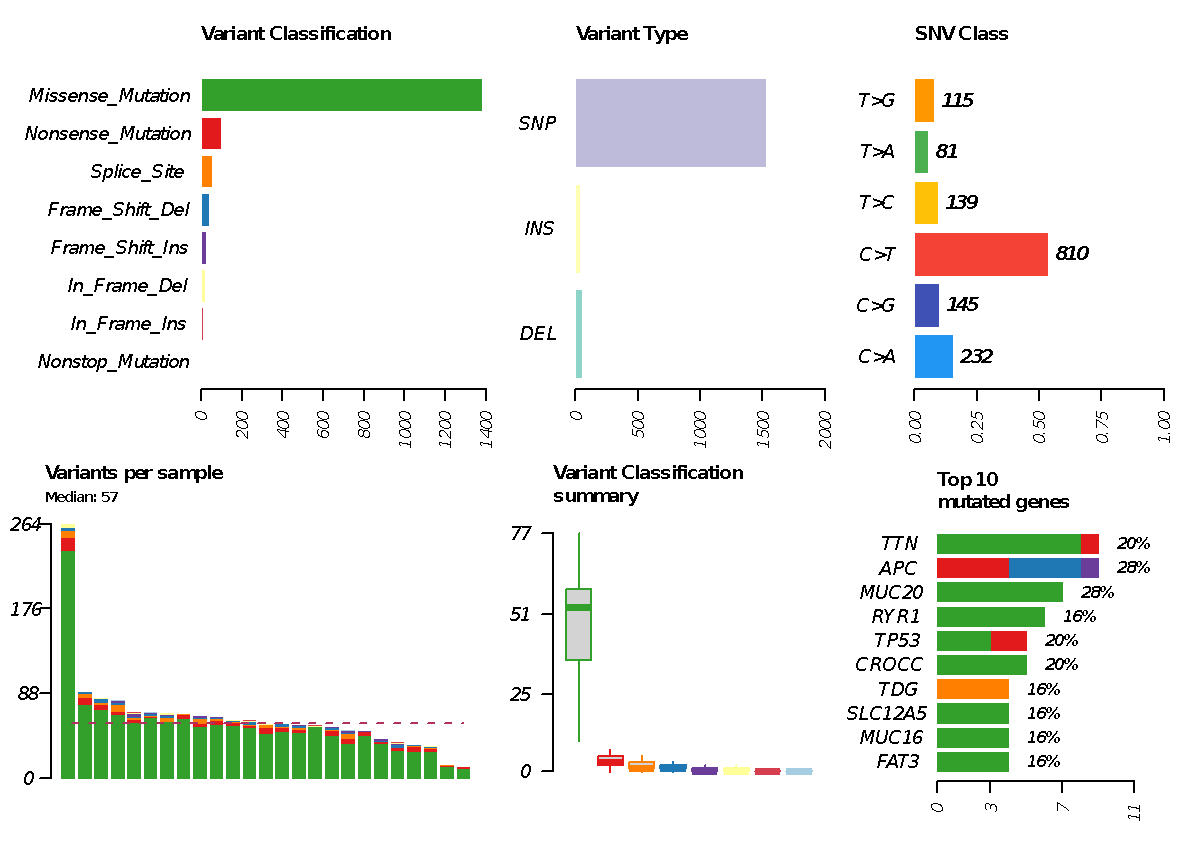


**(B)**


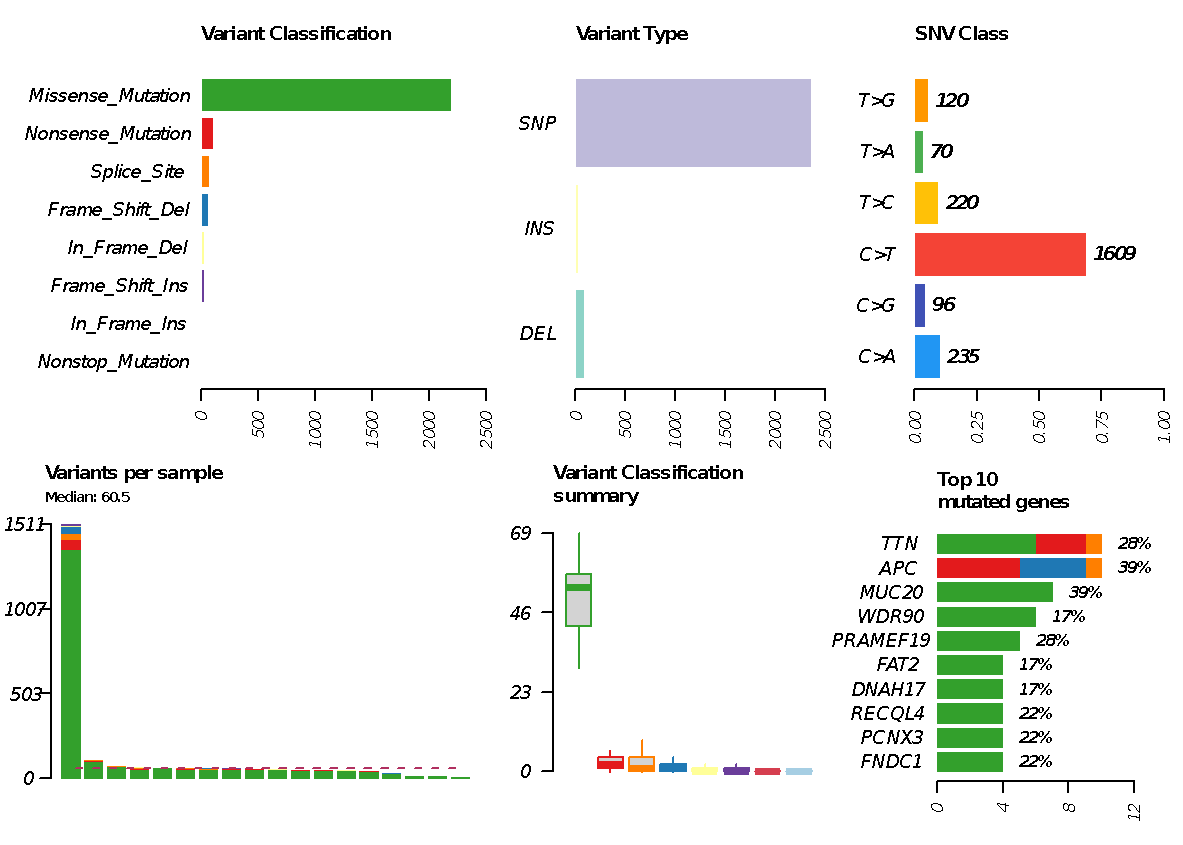


**Supplementary Figure S3. (A)** Mutational signatures identified by WES in Argentinian and Brazilian biopsies samples of locally advanced rectal cancer (LARC); **(B)** SBS5 mutational signature identified as differentially present in responder (R) and non-responder (NR) patients

**(A)**


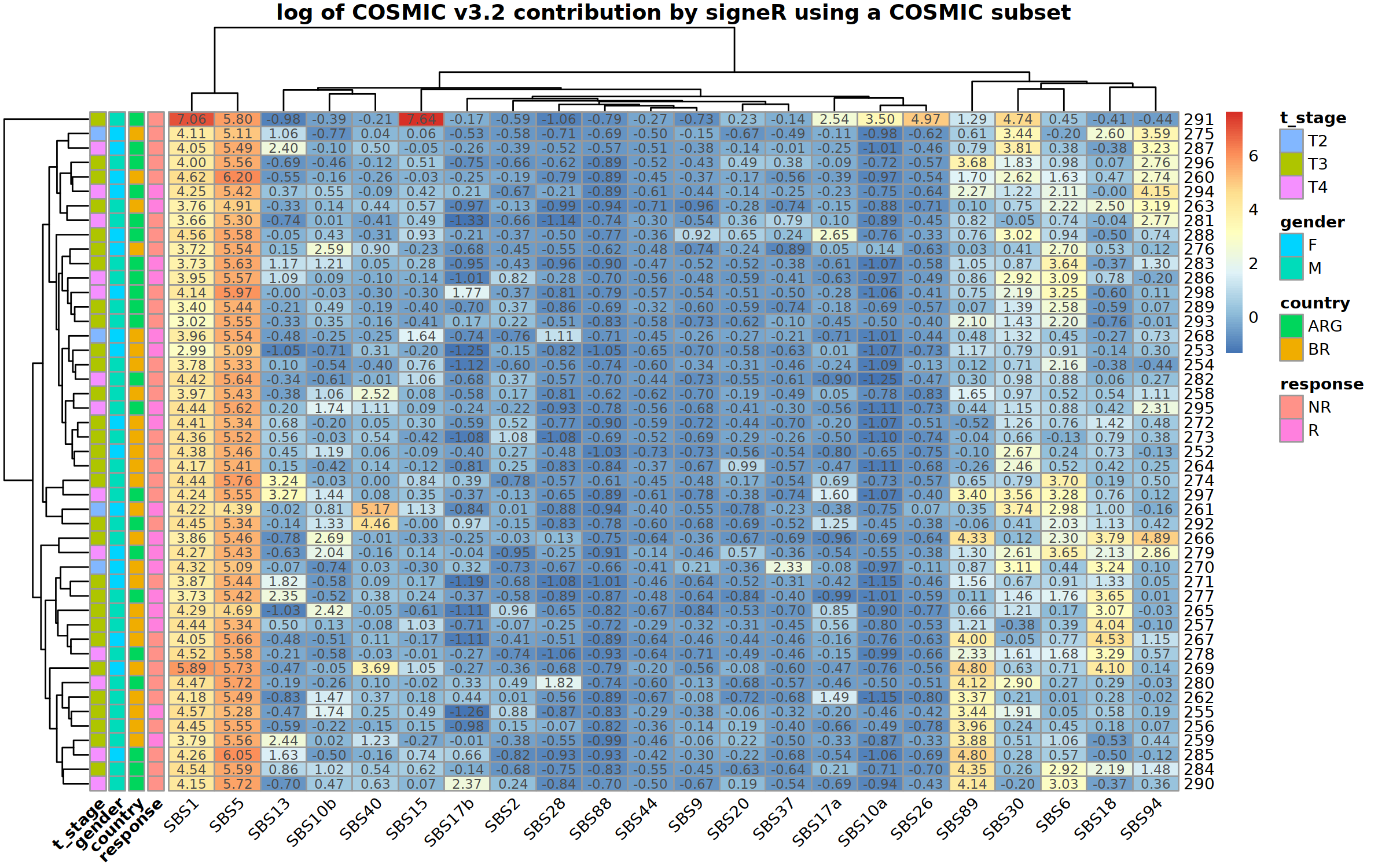


**(B)**

**
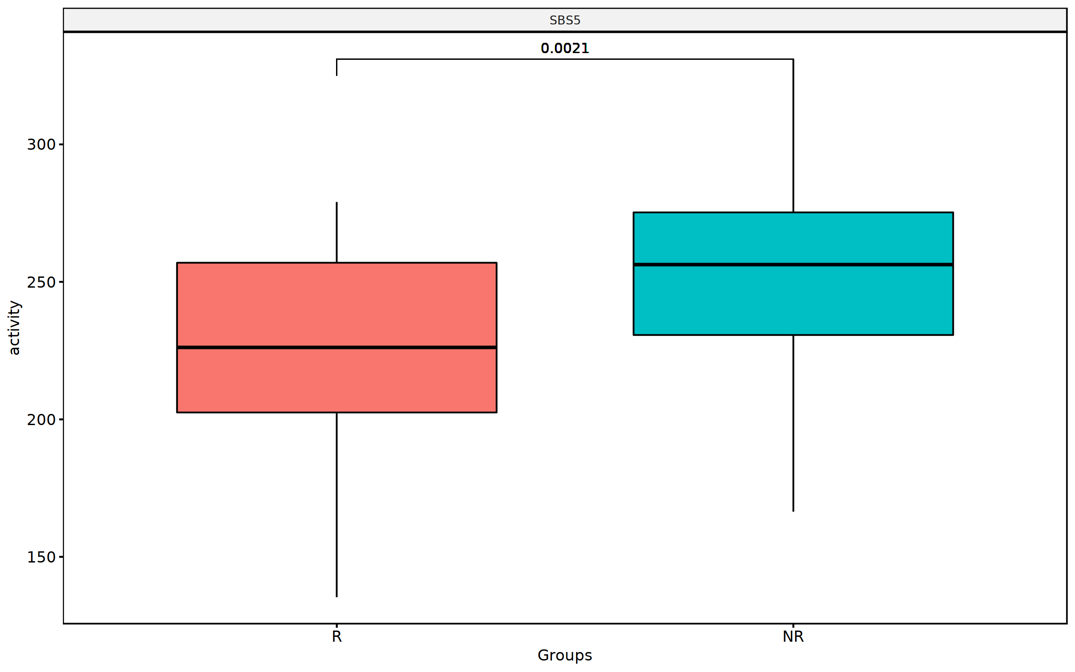
**

**Supplementary Figure S4.** Individual-based rarefaction curves for pre-treatment biopsies of locally advanced rectal cancer (LARC) obtained from Argentinian (blue) and Brazilian (red) patients. Sequences with at least 97% of similarity were clustered in operational taxonomic units (OTUs)

**
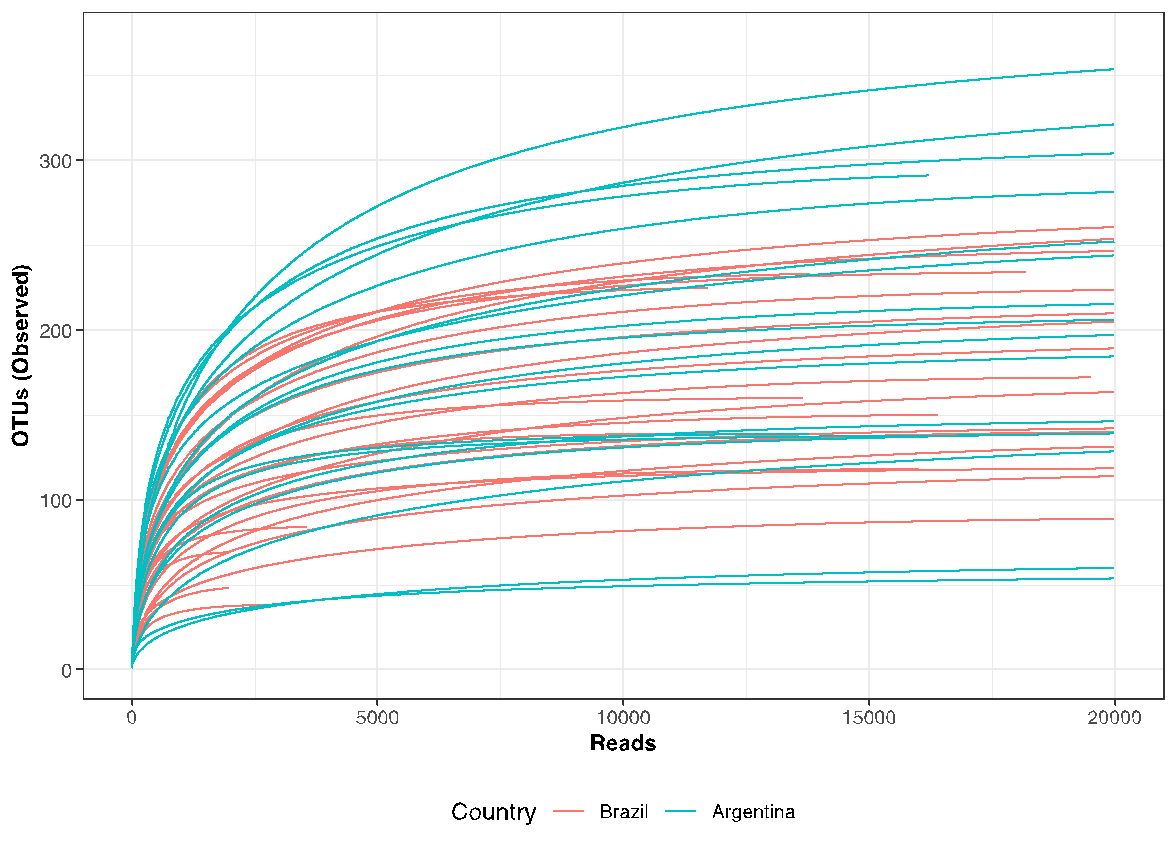
**

**Supplementary Figure S5.** Boxplots showing the bacterial alpha diversity of locally advanced rectal cancer (LARC) biopsies before neoadjuvant treatment using different metrics (Observed OTUs, Chao1, Shannon, Simpson indexes) between low and mid rectum tumor samples. No statistically significant differences were observed (Mann-Whitney U test *p-*value > 0.05)


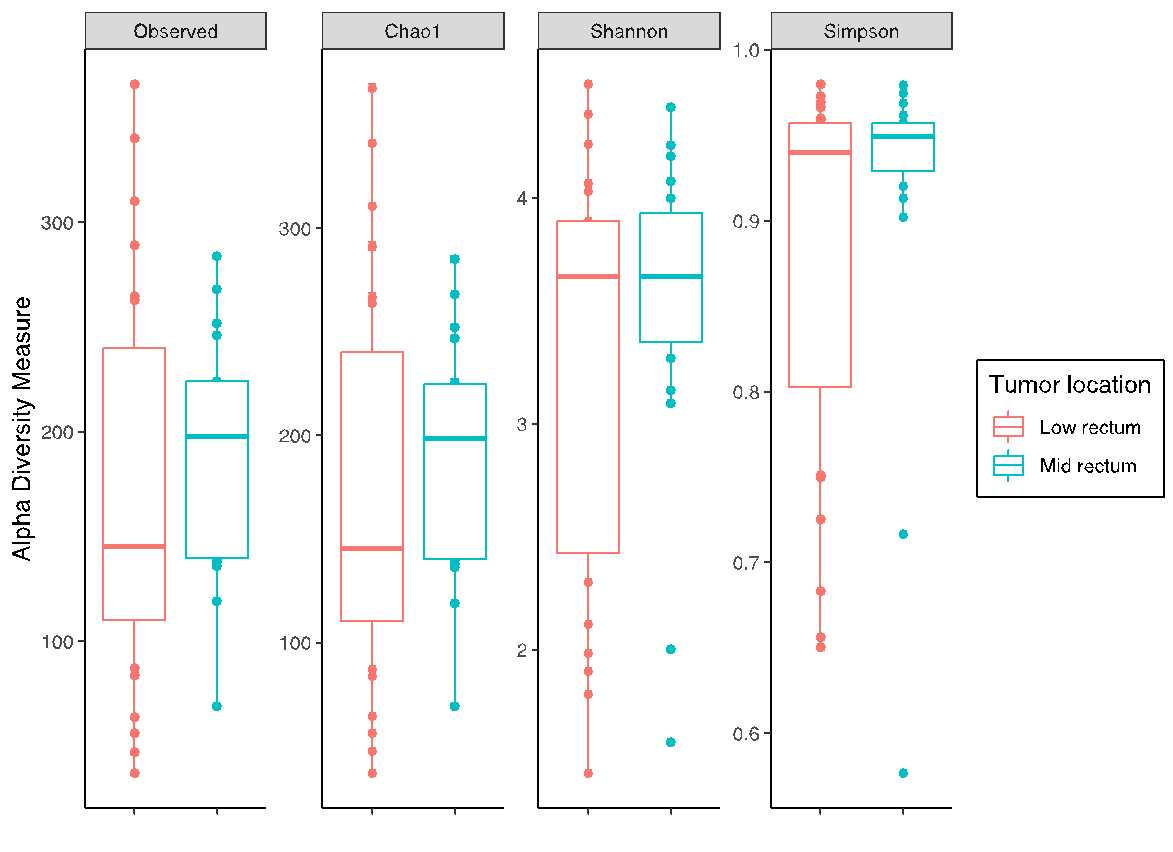


**Supplementary Figure S6.** Boxplots showing the bacterial alpha diversity of locally advanced rectal cancer (LARC) biopsies before neoadjuvant treatment using different metrics (Observed OTUs, Chao1, Shannon, Simpson indexes) between samples with different regression grades (CAP0 *vs.* CAP 1/2/3). No statistically significant differences were observed (Mann-Whitney U test *p-*value > 0.05)


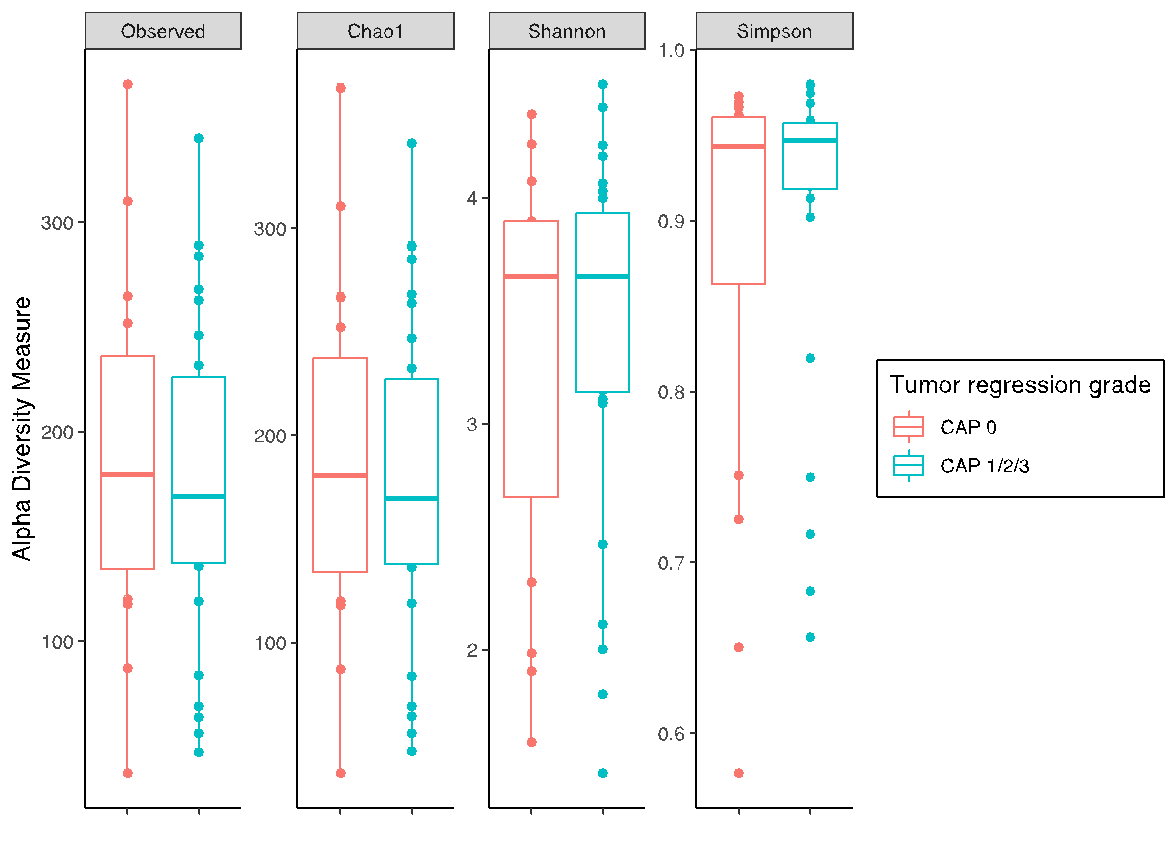


**Supplementary Figure S7.** Principal Coordinate Analysis (PCoA) ordination plots showing the bacterial beta diversity of locally advanced rectal cancer (LARC) biopsies before neoadjuvant treatment using three distances metrics (Bray-Curtis, Unweighted and Weighted UniFrac) comparing low and mid rectum tumor samples (PERMANOVA/ADONIS, *p-*value > 0.05)


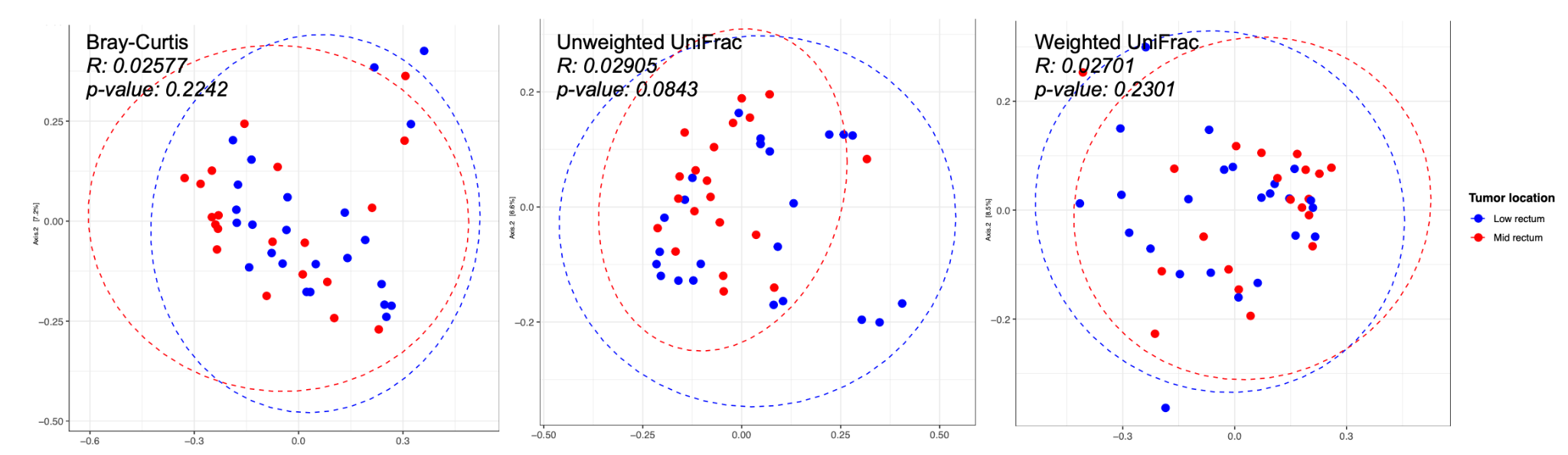


**Supplementary Figure S8.** Principal Coordinate Analysis (PCoA) ordination plots showing the bacterial beta diversity of locally advanced rectal cancer (LARC) biopsies before neoadjuvant treatment using three distances metrics (Bray-Curtis, Unweighted and Weighted UniFrac) between samples with different regression grades (CAP0 *vs.* CAP 1/2/3) (PERMANOVA/ADONIS, *p-*value > 0.05)

**
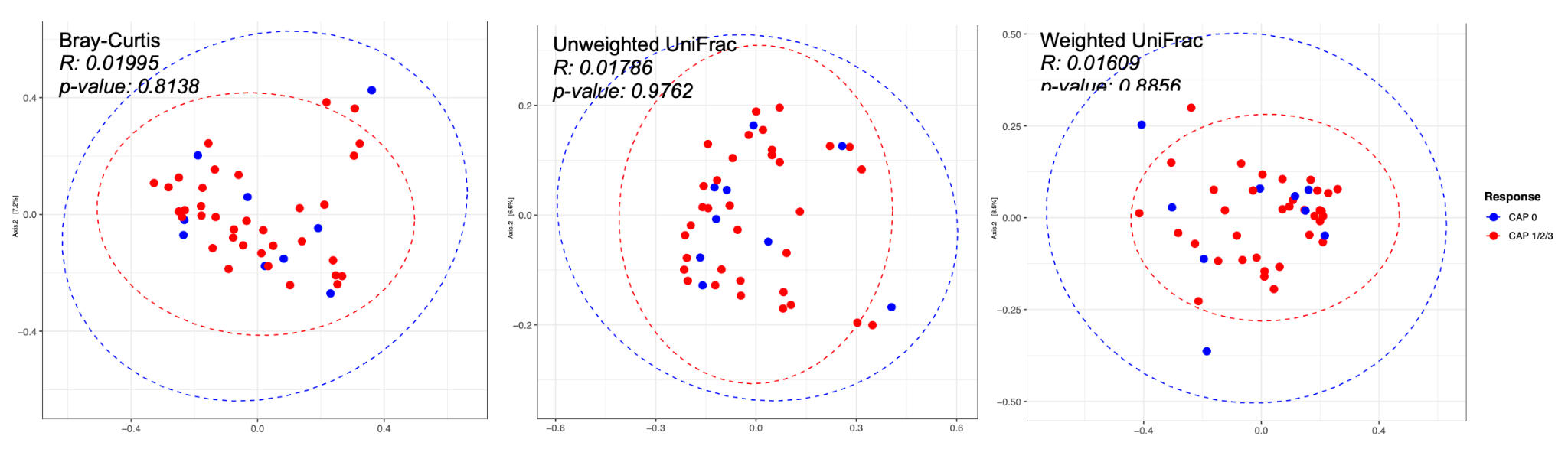
**
